# Polyphenolic Composition, Antioxidant and Antiproliferative Activity of Edible and Inedible Parts of Cultivated and Wild Pomegranate (*Punica granatum* L.)

**DOI:** 10.17113/ftb.61.04.23.8159

**Published:** 2023-12

**Authors:** Mirjana Milošević, Jelena Vulić, Zoran Kukrić, Biljana Lazić, Dragana Četojević-Simin, Jasna Čanadanović-Brunet

**Affiliations:** 1 University of Banja Luka, Faculty of Technology, Vojvode Stepe Stepanovića 75, 78000 Banja Luka, Bosnia and Herzegovina; 2 University of Novi Sad, Faculty of Technology, Bulevar cara Lazara 1, 21000 Novi Sad, Serbia; 3 Department of Pharmacy, Singidunum University, Danijelova 32, 11000 Belgrade, Serbia

**Keywords:** cultivated and wild pomegranate, phenolics, antioxidant activity, antiproliferative activity

## Abstract

**Research background:**

The aim of this study is to determine and compare the antioxidant and antiproliferative activities of juices and extracts of the peel, aril and membrane of the cultivated and wild pomegranate fruits.

**Experimental approach:**

The content of total phenols, total flavonoids, total flavonols, total flavan-3-ols and total anthocyanins was determined spectrophotometrically. The individual phenolics were quantified by HPLC. Antioxidant activity was determined by DPPH and ABTS tests and neutralisation of hydroxyl radical, while the antiproliferative activity was measured *in vitro* by sulforhodamine B (SRB) assay.

**Results and conclusions:**

Total phenolics were statistically highest in wild pomegranate peel extract, expressed in gallic acid equivalents, 340.92 mg/g (p<0.05), while total flavonoid content was the highest in cultivated pomegranate peel extract, expressed in quercetin equivalents, 31.84 mg/g (p<0.05). The sample of wild pomegranate peel extract showed the highest antioxidant activity with respect to free DPPH and ABTS radicals. The samples of cultivated pomegranate peel and membrane extracts had almost identical and the strongest effect on the inhibition of hydroxyl radicals (41.24 and 41.23 μg/mL, respectively). The sample of wild pomegranate peel extract showed the strongest effect on the growth inhibition of all tested tumour cell lines.

**Novelty and scientific contribution:**

In this study, the bioactivity of different parts of cultivated and wild pomegranates was determined and compared. In the available literature, the individual antioxidant and antiproliferative activity of only some parts of the pomegranate fruit was investigated. All parts of the pomegranate fruit were investigated, including the membrane, which was barely analysed in other works. The wild pomegranate has also been less analysed in previous studies. Future research should focus on *in vivo* studies of the obtained pomegranate samples.

## INTRODUCTION

Pomegranate (*Punica granatum* L.) fruit is one of the oldest edible fruits belonging to the family *Punicaceae*. This plant species originates from Asia, from the region from Iran to northern India. Pomegranates that grow in nature are known as wild pomegranates. The fruit of the wild pomegranate is smaller, while the fruit of the cultivated pomegranate is larger and heavier and has larger bright red grains (arils) of 8–12 mm in size. The pericarp, which contains many bioactive compounds such as flavonoids, ellagitannins and proanthocyanidins, makes up about 50 % of the total mass of pomegranate fruit. Arils, which are the edible part of the pomegranate, make up the remaining 50 % of the fruit mass. They are made up of an outer fleshy red part (78 %) and an inner seed (22 %) (*1*). Pomegranate fruit is divided into several cells (carpels) by membranous partitions (carpellary membranes), which are full of rounded succulent arils (*2*). It contains a large number of different phytochemicals. A total of around 50 polyphenols have been identified in different parts of the fruit. Pomegranate fruits contain hydrolysable tannins (punicalagin and punicalin), condensed tannins, anthocyanins, phenolic and organic acids. The concentration of bioactive compounds is the highest in the pomegranate bark (*3*). The pomegranate peel contains exceptional phytochemicals of medical and nutritional importance (*4*). Reactive oxygen species (ROS), formed during normal cellular metabolic processes or by exposure to ionising radiation or xenobiotic substances, are considered to be an important factor in the development of a large number of chronic diseases. The toxicity of ROS can be attributed to their ability to damage essential biological substrates, such as DNA, RNA, proteins and membrane lipids. ROS include superoxide radical, lipoperoxide oxides, hydrogen peroxide and hydroxyl radical. It is known that diet plays a key role in the prevention of many diseases. Due to the high content of polyphenolic components, pomegranate fruits are considered one of the foods whose antioxidants have numerous beneficial effects on human health. Phenolic compounds have the ability to scavenge free radicals and chelate metal cations. The aim and novelty of this study is to determine and compare the polyphenolic composition, antioxidant and antiproliferative activity of juices and extracts of all parts (peel, aril and carpellary membrane) of cultivated and little studied wild pomegranate fruits.

## MATERIALS AND METHODS

### Plant material

Ripe wild pomegranate fruits were harvested in November 2019 in Bosnia and Herzegovina, municipality of Stolac (43° 05′ N, 17° 58′ E). Samples of cultivated pomegranate fruits, originating from Turkey, were purchased at a local market in Banja Luka, Bosnia and Herzegovina in November 2019.

### Sample preparation

The peel, aril and membrane of cultivated and wild pomegranate fruits were manually separated from the fruit. Some of the arils were used to make pomegranate juice. After pressing the arils, the resulting juice was filtered and frozen at -18 °C. The remaining amount of arils was used to obtain aril extracts. Before extraction, they were crushed in a mortar with pestle. The peel and membrane were air-dried at room temperature for 20 days and then ground in a laboratory mill (E-1350 blender; Ema, Istanbul, Turkey).

### Determination of polyphenolic components

Powdered peels, arils and membranes (200 g) were extracted with 250 mL of solvent in a Soxhlet extractor (Intos Boral, Pula, Croatia). The extraction solvent was a mixture of *φ*(ethanol)=80 % (Zorka Pharma, Šabac, Serbia) and acetone (Lach-Ner s.r.o., Neratovice, Czech Republic) in *φ*(ethanol, acetone)=0.5. The extraction time was 6 h. After the extraction was completed, the extracts were evaporated to dryness in a rotary vacuum evaporator (Elektromedicina, Ljubljana, Slovenia) at 50 °C. The extracts were left in a vacuum desiccator in a dark place for 6 days to dry completely. The obtained dry extracts were kept at 4 °C until analysis. The samples were labelled as: CPJ for cultivated pomegranate juice, WPJ for wild pomegranate juice, CPPE for cultivated pomegranate peel extract, WPPE for wild pomegranate peel extract, CPAE for cultivated pomegranate aril extract, WPAE for wild pomegranate aril extract, CPME for cultivated pomegranate membrane extract and WPME for wild pomegranate membrane extract.

Total polyphenolic content was determined according to Kırca and Arslan (*5*) with certain modifications. Briefly, 0.2 mL of a diluted extract (juice) was mixed with 1 mL of 7.5 % NaHCO_3_ (Sigma-Aldrich, Merck, St. Louis, MO, USA) and 1.5 mL of 0.2 M Folin´s reagent (sodium 3,4-dioxo-3,4-dihydronaphthalene-1-sulfonate) (Sigma-Aldrich, Merck). After 30 min in a dark place, the absorbance was measured at 765 nm. The results were expressed on dry mass basis as mg gallic acid equivalents (GAE) per g extract and on fresh mass basis as mg GAE per g juice.

Total flavonoid content was determined according to Mohammed *et al*. (*6*) by mixing 2 mL of diluted sample with 2 mL of 2 % AlCl_3_ (Sigma-Aldrich, Merck) in 96 % ethanol. After 1 h at room temperature, the absorbance was measured at 420 nm. The results were expressed on dry mass basis as mg quercetin equivalents (QE) per g extract and on fresh mass basis as mg QE per g juice.

Total flavonol content was determined as described by Formagio *et al.* (*7*). The results were expressed on dry mass basis as mg QE per 1 gram of extract and on fresh mass basis as mg QE per g juice.

Total flavan-3-ol content was determined as previously described by Toro-Uribe *et al.* (*8*) with some modifications. Briefly, 2.5 mL of 10 % H_2_SO_4_ (Lach-Ner s.r.o.) in methanol (Lach-Ner s.r.o.) was mixed with 1 mL of diluted sample and 2.5 mL of 1 % vanillin (Sigma-Aldrich, Merck) in methanol. After 20 min at room temperature, the absorbance was measured at 500 nm. The results were expressed on dry mass basis as mg catechin equivalents (CE) per g extract and on fresh mass basis as mg CE per g juice.

Total anthocyanin content in samples was determined by the pH differential method according to Giusti and Wrolstad (*9*). Samples were extracted with HCl (Lach-Ner s.r.o.)/ethanol (*φ*(HCl, ethanol)=0.85:0.15) for 24 h at 0 °C and 0.5 mL extract was mixed with 9.5 mL HCl-KCl buffer (Sigma-Aldrich, Merck), pH=1.0. Absorbance was measured at 510 and 700 nm after 15 min of incubation at room temperature. The absorbance of anthocyanins was calculated as:

*A*=(*A*_510 nm_–*A*_700 nm_)_pH=1.0_ /1/

The total anthocyanin content (TAC/(mg/L)) of each sample was calculated using the following equation:

*c*(TAC)=(*A*·*M*·DF·1000)/(*ε*·*l*) /2/

where *A* is absorbance, *M* is the molar mass (449.2 g/mol), DF is the dilution factor (20), *ε* is the molar absorption coefficient of cyanidin-3-glucoside (26 900 L/(mol·cm)), and *l* is the length of the light path (1 cm). The anthocyanin concentration (mg/L) is then converted to mass fraction (mg/g). The results were expressed on dry mass basis as mg cyanidin-3-glucoside equivalent (CyGE) per g extract and on fresh mass basis also as mg CyGE per g juice.

### Identification and quantification of phenolic acids and flavonoids by HPLC method

Samples were analysed by a chromatographic system Shimadzu Prominence (Shimadzu, Kyoto, Japan). Chromatograms were recorded using different wavelengths for individual compounds: 280 and 320 nm for phenolic acids and 360 nm for flavonoids. Separation was performed on a Luna C-18 RP column, 5 mm, 250 mm×4.6 mm with a C18 guard column, 4 mm×30 mm (both from Phenomenex, Torrance, CA, USA). Two mobile phases, A (acetonitrile) and B (1 % formic acid), were used at a flow rate of 1 mL/min with the following gradient profile: 0–10 min from 10 to 25 % B, 10–20 min linear rise up to 60 % B and from 20 to 30 min linear rise up to 70 % B, followed by 10 min reverse to initial 10 % B with additional 5 min of equilibration time.

### Antioxidant activity

The antioxidant activities of the samples regarding 2,2-diphenyl-1-picrylhydrazyl (DPPH) radical and 2-azino-bis(3-ethylbenzothiazoline-6-sulfonic acid) (ABTS) radical were determined according to the methods of Liyana-Pathiranan and Shahidi (*10*) and Re *et al.* (*11*), respectively. The antioxidant capacities of samples to inhibit DPPH (Sigma-Aldrich, Merck) and ABTS (Sigma-Aldrich, Merck) radicals were presented as IC_50_ values (μg/mL).

Total antioxidant capacity to neutralise hydroxyl radicals (^•^OH) was determined by spectrophotometric method described by Xican (*12*). After keeping the samples at 50 °C for 20 min, 1 mL of 5 % trichloroacetic (Lach-Ner s.r.o.) and 1 % thiobarbituric acid (Sigma-Aldrich, Merck) were added. After mixing, all tubes were thermostated at 100 °C for 20 min. The samples were cooled to room temperature. The measurements were performed at 530 nm. The antioxidant capacity of samples to inhibit hydroxyl radical was presented as IC_50_ values (μg/mL).

### Antiproliferative effect

Human tumour cell lines: HeLa (cervix epitheloid carcinoma), MCF7 (breast adenocarcinoma), HT-29 (colon adenocarcinoma) and MRC-5 (normal fetal lung fibroblasts) were used for the estimation of cell growth activity.

Cell lines were harvested and plated into 96-well microtitre plates (Sarstedt, Newton, NC, USA) at seeding density of 4–8·10^3^ cells per well in a volume of 199 or 180 µL and preincubated in medium supplemented with 5 % foetal calf serum at 37 °C for 24 h. Serial dilutions of the samples and solvents as well as control (1 or 20 µL per well) were added to the test and control wells, respectively. Microplates were incubated at 37 °C for an additional 48 h. Cell growth was assessed using colourimetric sulforhodamine B (SRB) assay according to Cetojevic-Simin *et al.* (*13*). Effects on cell growth were calculated as:

Cell growth = ((*A*_t_/*A*_c_)·100 /3/

where *A*_t_ is the absorbance of the test sample and *A*_c_ is the absorbance of the control.

All spectrophotometric analyses were determined by UV-VIS spectrophotometer (Jenway® 6305; Cole-Parmer, St Neots, UK).

### Statistical analysis

The experiments were carried out in at least three repetitions. The results were expressed as mean value±standard deviation (S.D.). All results were subjected to a one-factor analysis of variance (ANOVA). The Duncan's test was performed to determine a statistically significant difference between the arithemtic means at p<0.05. The results were obtained using the software programs: Microsoft Excel (*14*), Origin 5.0 (*15*) and Statistica 12.0 (*16*).

## RESULTS AND DISCUSSION

The obtained juice yields were: for cultivated pomegranate juice (CPJ) 80.51 % and for wild pomegranate juice (WPJ) 48.73 %. Higher yield of CPJ can be explained by the higher amount of liquid phase in the arils of the cultivated pomegranate fruit. The yield of cultivated pomegranate juice was in accordance with the studies of Zaouay *et al.* (*17*). The yields of cultivated pomegranate peel extract (CPPE), cultivated pomegranate aril extract (CPAE) and cultivated pomegranate membrane extract (CPME) were: 17.83, 11.01 and 22.27 %, respectively. Yields of wild pomegranate peel extract (WPPE), wild pomegranate aril extract (WPAE) and wild pomegranate membrane extract (WPME) were: 14.05, 13.26 and 40.20 %, respectively. Yield of CPPE in this study was similar to the results reported by Iqbal *et al.* (*18*), whose yield of ethanol extract of pomegranate peel was 21.14 %. The yield of cultivated pomegranate aril extract was lower than that obtained by Magangana *et al.* (*19*).

The mass fraction of total polyphenolics was statistically highest in the WPPE sample (p<0.05). The CPPE sample had a slightly lower mass fraction of these components, with a statistically significant difference compared to the WPPE sample (p<0.05). The lowest mass fraction of these compounds was in the CPJ sample, which did not differ statistically significantly from the WPJ, CPAE and WPAE samples (Table 1). Value for the CPPE sample (295 mg/g) was in accordance with the results reported by Derakhshan *et al.* (*20*) for Natanz pomegranate peel (276 mg/g). On the other hand, Orak *et al.* (*21*) reported about twice as low mass fraction of total phenols in ethanolic extracts of pomegranate peel of the Hicaznar variety. Our values for wild and cultivated pomegranate juices were in accordance with the values published by Gözlekçi *et al.* (*22*), which were in the range of 0.78–1.55 mg/g and slightly lower for CPJ sample than in the study by Zaouay *et al.* (*17*). The mass fraction of total phenols in the CPAE sample was slightly higher than the literature values for ethanolic extracts of arils of different varieties of pomegranate (3.2−8.8 mg/g) (*19*). The mass fraction of total phenols in the WPME sample was statistically higher than in the CPME sample (p<0.05). The mass fraction of total flavonoids was the highest in the CPPE sample and it was statistically significantly different from the WPPE sample (p<0.05). A similar trend was observed in the pomegranate carpellary membrane, where the mass fraction of these compounds in CPME was statistically higher than in the WPME sample (p<0.05). The lowest mass fraction of these compounds was observed in the CPJ and WPJ samples (0.12 and 0.13 mg/g, respectively). The WPJ sample had a slightly higher mass fraction of these compounds, but without a statistically significant difference (p>0.05). Since the juice was obtained by pressing of arils and is their integral part, the WPAE sample was also richer in these compounds than CPAE and the difference was statistically significant (p<0.05). The mass fraction of flavonoids in CPJ was consistent with the results obtained by Li *et al.* (*23*), whose concentrations were in the range of 0.045–0.335 mg/mL depending on the pomegranate variety. The mass fraction of total flavonoids in the CPPE sample was 2 to 3 times higher than the literature values (*21*, *24*). Taking the above data into account, we can conclude that flavonoids are mainly located in the peel and membrane of pomegranate fruits, while their content in the arils and pomegranate juice is significantly lower.

**Table 1:** Mass fraction of total polyphenolics, total flavonoids, total flavonols, total flavan-3-ols and total anthocyanins in cultivated and wild pomegranate juices and extracts

Sample	*w*(total phenols as GAE)/(mg/g)	*w*(total flavonoids as QE)/(mg/g)	*w*(total flavonols as QE)/(mg/g)	*w*(total flavonols as CE)/(mg/g)	*w*(total anthocyanins as CyGE)/(mg/g)
CPJ	(0.85±0.03)^a^	(0.12±0.00)^a^	(1.56±0.01)^a^	(6.9±0.7)^a,b^	(0.45±0.01)^c^
WPJ	(1.84±0.02)^a^	(0.13±0.00)^a^	(1.61±0.04)^a^	(14.6±0.8)^b^	(0.53±0.01)^d^
CPAE	(6.6±0.2)^a^	(0.52?±0.006)^b^	(6.94±0.05)^a^	(10.5±0.7)^a,b^	(0.24?±0.006)^a^
WPAE	(16.2±1.4)^a^	(1.38±0.02)^c^	(18.1±0.5)^b^	(11.1±0.8)^a,b^	(0.42±0.00)^c^
CPPE	(295±204)^b^	(31.8±0.2)^d^	(259±7)^c^	(76.1±11.4)^c^	(1.68±0.01)^e^
WPPE	(341±26)^c^	(29.8±0.2)^e^	(287.4±0.6)^d^	(40.0±1.7)^d^	(0.85±0.05)^f^
CPME	(155.4±5.0)^d^	(21.6±0.4)^f^	(144.1±7.8)^e^	(6.4±0.2)^a^	(0.58±0.006)^g^
WPME	(201±11)^e^	(18.36±0.04)^g^	(154.1±0.4)^f^	(8.97±0.00)^a,b^	(0.37±0.02)^b^

The results are presented as mean value±standard deviation (*N*=3). Mean values with different letters in superscript in the same column are statistically diferent (p<0.05). CPJ=cultivated pomegranate juice, WPJ=wild pomegranate juice, CPPE=cultivated pomegranate peel extract, WPPE=wild pomegranate peel extract, CPAE=cultivated pomegranate aril extract, WPAE=wild pomegranate aril extract, CPME=cultivated pomegranate membrane extract and WPME=wild pomegranate membrane extract, GAE=gallic acid equivalent, QE=quercetin equivalent, CE=catechin equivalent, CyGE=cyanidin-3-glucoside equivalent

The mass fraction of total flavonols was statistically highest in the WPPE sample and was slightly higher than in CPPE with a statistically significant difference (p<0.05). Similar results were observed in the WPME and WPAE samples, which had a statistically higher mass fraction than CPME and WPAE samples (p<0.05). CPJ had the lowest mass fraction of flavonols, and their content was similar to WPJ and CPAE samples, without a statistically significant difference (p>0.05). The mass fractions of total polyphenolic compounds and total flavonols were higher in all samples of wild pomegranate fruit than in the samples of cultivated pomegranate fruit. Furthermore, it can be concluded from the presented results that the total phenols, flavonoids and anthocyanins are mainly distributed in the outer part of the fruit (peel) and less in the interior, which is in agreement with the research of Saidani *et al.* (*25*).

The CPPE sample showed statistically highest content of total flavan-3-ols, followed by the WPPE sample, which had about twice as low amounts of these compounds. The CPJ sample was the poorest in these substances and did not differ statistically significantly from the CPAE, WPAE and WPME samples (p>0.05). The CPJ also had about twice as low mass fraction of total flavan-3-ol compared to WPJ (p>0.05). The WPAE sample had a slightly higher mass fraction of these compounds than CPAE (p<0.05). The WPME sample also had a higher mass fraction of total flavan-3-ol and was statistically different from the CPME sample (p<0.05).

The mass fraction of total anthocyanins was statistically highest in the WPJ sample and the lowest in the WPME sample. Compared with the CPJ sample, WPJ and WPAE samples had a statistically higher mass fraction of these compounds. The CPJ sample had a higher content of total anthocyanins than that obtained by Li *et al.* (*23*) (0.026–0.160 mg/mL). The mass fraction of total anthocyanins in the CPAE and CPPE samples was significantly lower than the results of Osama *et al.* (*26*) (11.04 and 15.24 mg/g, respectively). The mass fraction of total anthocyanins was statistically higher in CPPE than in WPPE, and higher in the CPME than in the WPME sample, with a statistically significant difference (p<0.05). The results of this study in relation to the CPPE sample were lower than the values reported by Fawole *et al.* (*27*), which ranged from 0.058 to 0.32 mg/g depending on the variety of pomegranate fruit. It can be concluded from the above data that these compounds are stored slightly more in the outer part of the fruit, in contrast to the wild pomegranate fruits, where the anthocyanins are located more inside the fruit.

The contents of individual phenolic compounds are shown in Table 2. The highest total mass fraction of phenolic compounds detected by HPLC method was in the WPPE sample (46.61 mg/g), closely followed by CPPE (42.40 mg/g) and WPME (40.92 mg/g). The highest mass fractions of epicatechin (9.53 mg/g), *p*-hydroxybenzoic (9.24 mg/g), syringic (4.38 mg/g), ellagic (1.23 mg/g) and ferulic (1.97 mg/g) acids were in WPPE sample. The same sample showed the best results in antioxidant (DPPH, ABTS and OH radicals) tests (Table 3). Epicatechin gallate was found in the peel and membrane (CPPE, WPPE, CPME and WPME) samples. Gallic acid was found in the highest amount in CPPE (3.58 mg/g), as well as coumaric (3.65 mg/g) and chlorogenic (6.15 mg/g) acids. The CPPE sample followed closely the WPPE sample in good antioxidant results. Protocatechuic acid was highest in the CPME sample (4.98 mg/g), which is consistent with the slightly poorer results of the bioactive tests in Table 3 than in the peel samples. Vanillic acid was detected only in the WPPE and WPME samples.

**Table 2:** Mass fraction of phenolic compounds in the samples of cultivated and wild pomegranate fruits

Phenolic compound	*w*/(mg/g)							
CPJ	WPJ	CPAE	WPAE	CPPE	WPPE	CPME	WPME
Gallic acid	(0.11±0.03)	(0.20±0.02)	(0.15±0.01)	(0.44±0.02)	(3.58±0.04)	(1.39±0.03)	(1.54±0.03)	(2.65±0.02)
Protocatechinic acid	(0.12±0.03)	(0.05±0.02)	(0.22±0.02)	(0.61±0.02)	(4.25±0.02)	(1.97±0.02)	(4.98±0.02)	(2.47±0.03)
Epicatechin	(0.24±0.03)	(0.28±0.02)	(0.21±0.01)	0	(3.77±0.03)	(9.53±0.03)	(7.24±0.03)	(6.93±0.02)
Catechin	0	0	(0.74±0.01)	(3.27±0.01)	(6.18±0.02)	(1.65±0.02)	(6.22±0.02)	(8.93±0.03)
Ferulic acid	(0.01±0.01)	(0.01±0.01)	(0.04±0.01)	(0.09±0.02)	(1.15±0.02)	(1.97±0.02)	(0.81±0.01)	(1.11±0.02)
Syringic acid	0	0	0	0	(3.46±0.02)	(4.38±0.01)	(1.13±0.01)	(2.84±0.01)
Ellagic acid	0	0	0	0	(0.42±0.01)	(1.23±0.01)	(0.14±0.02)	(0.17±0.02)
Coumaric acid	0	0	0	0	(3.65±0.02)	(2.60±0.02)	(1.63±0.02)	(2.98±0.02)
Chlorogenic acid	(0.01±0.00)	(0.01±0.00)	(0.04±0.01)	(0.83±0.01)	(6.15±0.01)	(4.48±0.02)	(2.80±0.02)	(4.36±0.02)
*p*-hydroxybenzoic acid	(0.24±0.02)	(0.46±0.02)	0	0	(5.04±0.01)	(9.24±0.02)	(3.12±0.02)	(3.96±0.02)
Vanillic acid	0	0	0	0	0	(3.23±0.02)	0	(1.15±0.02)
Epicatechin gallate	0	0	0	0	(4.76±0.01)	(4.94±0.02)	(1.60±0.02)	(3.37±0.02)
Total	0.73	1.01	1.40	5.24	42.40	46.61	31.22	40.92

The results are presented as mean value±standard deviation (*N*=3). CPJ=cultivated pomegranate juice, WPJ=wild pomegranate juice, CPAE=cultivated pomegranate aril extract, WPAE=wild pomegranate aril extract, CPPE=cultivated pomegranate peel extract, WPPE=wild pomegranate peel extract, CPME=cultivated pomegranate membrane extract and WPME=wild pomegranate membrane extract

**Table 3:** Antioxidant activity of cultivated and wild pomegranate juices and extracts

Sample	DPPH test	ABTS test	Hydroxyl radical
IC_50_/(μg/mL)		
CPJ	(5088±418)^d^	(1252±68)^b^	(9313±1612)^a^
WPJ	(1956±178)^c^	(478±26)^c^	(4121±645)^b^
CPAE	(599±19)^b^	(148.7±5.6)^d^	(659±109)^c^
WPAE	(217±19)^a^	(67.0±1.4)^e^	(445±60)^c^
CPPE	(14.4±0.7)^a^	(3.5±0.2)^a^	(41.2±4.6)^c^
WPPE	(12.2±0.7)^a^	(3.2±0.1)^a^	(36.4±4.9)^c^
CPME	(21.0±1.0)^a^	(5.8±0.3)^a^	(41.2±2.5)^c^
WPME	(16.9±079)^a^	(4.7±0.3)^a^	(37.9±2.8)^c^
Trolox	(12.6±0.7)	(3.0±0.)	(4.4±0.4)

The results are presented as mean value±standard deviation (*N*=3). Mean values with different letters in superscript in the same column are statistically diferent (p<0.05). CPJ=cultivated pomegranate juice, WPJ=wild pomegranate juice, CPAE=cultivated pomegranate aril extract, WPAE=wild pomegranate aril extract, CPPE=cultivated pomegranate peel extract, WPPE=wild pomegranate peel extract, CPME=cultivated pomegranate membrane extract and WPME=wild pomegranate membrane extract

The WPPE sample had the highest antioxidant activity against free DPPH and ABTS radicals, followed by the CPPE, WPME and CPME samples, with no statistically significant difference (p>0.05) (Table 3). On the other hand, CPJ showed the lowest antioxidant activity against DPPH and ABTS free radicals, while the WPJ had about 2.6 times higher acitivity than the CPJ sample. All samples showed that the components of the wild pomegranate have a higher antioxidant capacity than those of the cultivated pomegranate fruit. Sharayei *et al.* (*28*) reported significantly lower antioxidant activity of pomegranate peel extract obtained by aqueous extraction assisted by ultrasonic waves, whose IC_50_ values obtained with DPPH test ranged from 0.2 to 1.2 mg/mL. On the other hand, Okonogi *et al.* (*29*) reported for the same DPPH test IC_50_=3 μg/mL in their study with pomegranate peel extract in 95 % ethanol. A similar result was reported by Kanatt *et al.* (*30*) (IC_50_=4.9 μg/mL). Our results were consistent with the values obtained by Mansour *et al*. (*31*) for aqueous extract of pomegranate peel, where IC_50_ values were in the range of 10.2−13.1 μg/mL. Robert *et al.* (*32*) reported for pomegranate juice IC_50_=2.12 mg/mL, which means that this sample showed about two times better antioxidant activity than our CPJ sample, but also slightly worse than our sample WPJ. The CPAE and WPAE samples showed significantly better antioxidant activity than the literature data (IC_50_=1.73 mg/mL for ethanol extract) (*32*).

Regarding the ABTS radical, the CPJ sample showed the results in agreement with those found in the literature (IC_50_=525−3760 μg/mL) (*33*). Also, the extract of ethanolic arils obtained by Singh *et al.* (*34*) showed better antioxidant activity than our CPAE sample, but worse than the WPAE sample (IC_50_=81.31 μg/mL). The same authors confirmed the significant influence of solvent choice during the extraction on the antioxidant activity of the extracts. The CPPE sample had significantly better activity than the aqueous extract in the work of Al-Hindi and Abd El Ghani (*35*) (IC_50_=54.63 μg/mL). Likewise, our peel extracts had better activity than the results reported by Laosirisathian *et al*. (*36*) for pomegranate peel extracts at different volume fractions of ethanol as solvent.

Similar to the DPPH and ABTS tests, all samples of wild pomegranate fruit showed better antioxidant activity against hydroxyl radicals. The CPPE and CPME samples showed almost identical and the strongest effect on hydroxyl radical inhibition, as did the WPPE and WPME samples. The mean values of these samples did not differ statistically significantly (p>0.05). The CPJ sample had the weakest antioxidant activity compared to the other samples and was statistically significantly different from all other samples (p<0.05). The WPAE sample showed stronger antioxidant activity against the OH radical than the CPAE sample, but with no statistically significant difference (p>0.05). Our results for pomegranate peel extracts were slightly better than the results of Arun *et al*. (*37*), whose IC_50_ value for pomegranate peel extract in 70 % methanol was (54.9±0.4) μg/mL, while according to the same authors, the IC_50_ value for the peel extract obtained with pure methanol was (13.6±0.3) μg/mL. Our peel extracts showed better activity than the study by Rummun *et al.* (*38*), where IC_50_ of the methanolic peel extract was 0.111 mg/mL.

To date, several studies have been conducted to investigate the antiproliferative activity of pomegranate fruit against different types of tumour cells, such as colon, breast, prostate, lung and cervical cancer (*39*). The published study (*40*) showed that ellagic acid and its by-products can contribute to the prevention of colon cancer by regulating the expression of multiple genes involved in key processes of cancer development. A study of 46 patients with experimental prostate cancer showeda significant reduction in prostate-specific antigen (PSA) in 16 of them during treatment with pomegranate juice (*41*).

Table 4 shows that the CPJ and WPJ samples did not show antiproliferative activity on the tested tumour cells. In general, the WPPE sample showed the strongest effect on the growth inhibition of all tested tumour cell lines compared to all other samples. Based on the research by Abdel Motaal and Shaker (*41*), our WPPE sample showed significantly lower antiproliferative activity on MCF7 cells (IC_50_=(7.70±0.01) μg/mL). Keta *et al.* (*40*) also reported about 2.5 times better antiproliferative activity than our WPPE sample on MCF7 cells (IC_50_=(31.29±1.63) μg/mL). With the exception of the CPJ and WPJ samples, the weakest antiproliferative effect on the tested tumour cell lines was observed in the CPAE and WPAE samples. The CPME sample showed a better antiproliferative effect than the CPPE and WPME samples in terms of the inhibition of HeLa and MCF7 cells. The CPPE sample showed better antiproliferative activity than WPME. Authors Peršurić *et al*. (*43*) reported IC_50_ values for the antiproliferative activity of pomegranate extracts on HeLa cells in the range of 0.141–0.212 mg/mL. Comparing the obtained IC_50_ values for antiproliferative activity on HT-29 cells, the CPPE sample showed a better inhibitory effect than the WPME and CPME samples. For the HT-29 tumour cell line, the WPME sample showed a better effect than the CPME sample. For the MRC-5 tumour cell line, the CPPE sample showed slightly better antiproliferative activity than the CPME and WPME samples, while both CPME and WPME samples showed a similar effect in inhibiting the growth of these tumour cell lines. The WPPE sample showed slightly better antiproliferative activity on the MRC-5 tumour cell line than the studies by Keta *et al.* (*42*), who reported an IC_50_=(189.15±0.05) μg/mL.

**Table 4:** Antiproliferative effects of cultivated and wild pomegranate juices and extracts on HeLa (cervix epitheloid carcinoma), MCF7 (breast adenocarcinoma), HT-29 (colon adenocarcinoma) and MRC-5 (normal fetal lung fibroblasts) cells

Sample	HeLa	MCF7	HT-29	MRC-5
	IC_50_/(μg/mL)		
CPJ	n.a.	n.a.	n.a.	n.a.
WPJ	n.a.	n.a.	n.a.	n.a.
CPAE	>5000^a^	>5000^a^	>5000^a^	>5000^a^
WPAE	(4120±163)^b^	(3355±146)^b^	(4745±94)^a^	(4387±156)^a^
CPPE	(273±55)^c^	(139.6±7.8)^c^	(550±95)^b^	(209.6±10.4)^b^
WPPE	(74.6±23.3)^d^	(85.8±25.8)^d^	<312.5^c^	(145.2±11.0)^c^
CPME	(157±54)^e^	(124.2±23.7)^c^	(2060±112)^d^	(297±12^b^
WPME	(438±77)^f^	(153.5±5.7)^c^	(1445±83)^e^	(300±25)^b^

The values represent mean value±standard deviation (*N*=4). n.a.=no activity. Mean values with different letters in superscript in the same column are statistically diferent (p<0.05). CPJ=cultivated pomegranate juice, WPJ=wild pomegranate juice, CPAE=cultivated pomegranate aril extract, WPAE=wild pomegranate aril extract, CPPE=cultivated pomegranate peel extract, WPPE=wild pomegranate peel extract, CPME=cultivated pomegranate membrane extract and WPME=wild pomegranate membrane extract

## CONCLUSIONS

Extracts from wild pomegranate peel contained the highest amounts of total phenols and flavonols, while the highest mass fraction of flavonoids, flavan-3-ol and total anthocyanins was found in the extract of cultivated pomegranate peel. The extract of wild pomegranate peel also showed the highest antioxidant activity against DPPH, ABTS and hydroxyl radicals compared to the other tested pomegranate samples. The same sample showed the highest antiproliferative activity on the tested tumour cell lines. As can be observed, the total phenols, flavonoids and flavonols have a very strong effect on the antiproliferative activity of the pomegranate samples against MCF7, HT-29 and MRC-5 tumour cell lines. In this study, all parts of the pomegranate fruits, cultivated and wild, including the membrane of the pomegranate, were analysed and ethanol, which is a green solvent, was used for the extraction. Future studies with the obtained pomegranate samples should be performed *in vivo*.
